# Restructuring of Lamina-Associated Domains in Senescence and Cancer

**DOI:** 10.3390/cells11111846

**Published:** 2022-06-05

**Authors:** Aurélie Bellanger, Julia Madsen-Østerbye, Natalia M. Galigniana, Philippe Collas

**Affiliations:** 1 Department of Molecular Medicine, Institute of Basic Medical Sciences, Faculty of Medicine, University of Oslo, 0372 Oslo, Norway; a.n.p.bellanger@medisin.uio.no (A.B.); j.k.madsen-osterbye@medisin.uio.no (J.M.-Ø.); n.m.galigniana@medisin.uio.no (N.M.G.); 2 Department of Immunology and Transfusion Medicine, Oslo University Hospital, 0372 Oslo, Norway

**Keywords:** cancer, EMT, epigenome, heterochromatin, lamin, lamina-associated domain, senescence

## Abstract

Induction of cellular senescence or cancer is associated with a reshaping of the nuclear envelope and a broad reorganization of heterochromatin. At the periphery of mammalian nuclei, heterochromatin is stabilized at the nuclear lamina via lamina-associated domains (LADs). Alterations in the composition of the nuclear lamina during senescence lead to a loss of peripheral heterochromatin, repositioning of LADs, and changes in epigenetic states of LADs. Cancer initiation and progression are also accompanied by a massive reprogramming of the epigenome, particularly in domains coinciding with LADs. Here, we review recent knowledge on alterations in chromatin organization and in the epigenome that affect LADs and related genomic domains in senescence and cancer.

## 1. Introduction

Spatial chromatin organization in the mammalian nucleus is under the influence of extracellular stimuli and is a crucial regulator of gene expression programs. Induction of cellular senescence, a state characterized by an inability to re-enter the cell cycle, and cancer, which requires cell proliferation, interferes with normal cell fate and tissue homeostasis. Senescence and cancer are functionally connected, as senescence is implicated in cancer prevention by causing cell division arrest, and in cancer aggressiveness through the secretion of bioactive compounds [1,2].

Induction of senescence and cancer elicits massive rearrangements in the epigenome and in chromatin architecture. The latter includes a re-wiring of short- and long-range chromosomal interactions and of associations of chromosomes with the nuclear lamina (NL), at the nuclear periphery, via a repositioning of lamina-associated domains (LADs). Cancer cells, in addition, display genomic rearrangements that have consequences on three-dimensional (3D) and radial (center-to-periphery) chromatin organization, which are reinforced by epigenetic alterations of DNA and histones. Genomic and 3D chromatin changes resulting from senescence and cancer initiation have been recently reviewed elsewhere (senescence [3,4,5,6]; cancer [7,8,9]) and are not addressed here.

Increasing evidence indicates that the nuclear periphery is a dynamic compartment and plays a role in the establishment of high-order senescence and cancer chromatin phenotypes. Here, we adopt a nuclear sideline view of chromatin and address current knowledge on chromatin structural changes and epigenetic alterations affecting LADs and related domains in senescence and cancer.

## 2. Chromatin Organization at the Nuclear Periphery: Consistency and Variation in LADs

The 3D organization of chromatin is able to change in response to external signals during development, differentiation, and tissue homeostasis. This view rests on discoveries of genomic elements and architectural proteins that structurally shape the genome at multiple levels [10,11,12]. At the largest scale, chromosomes are organized in territories broadly divided into lose, euchromatic, and transcriptionally active A compartments, and more compact heterochromatic and largely repressed B compartments enriched in LADs. Within compartments, topological domains of higher chromatin contact frequencies are important for gene expression regulation, and establish long-range contacts with other topological domains, shaping higher-order chromatin architecture [13,14,15]. 

At the nuclear periphery, chromatin is stabilized by interactions with the nuclear envelope, an outer and inner nuclear membrane system interfacing chromatin via the nuclear lamina (NL). The NL is a meshwork intermediate filaments of A-type lamins (lamin A/C or LMNA/C), splicing products of the *LMNA* gene, and B-type lamins (lamins B1 and B2), encoded by the *LMNB1* and *LMNB2* genes [16]. The NL dynamically sequesters signaling molecules, chromatin modifiers, and transcription factors and imposes a spatio-temporal regulation of DNA replication and transcription [17]. Interactions of chromatin with the NL occur through hundreds of LADs unevenly distributed throughout the genome [18,19]. LADs are a conserved feature of genome organization in virtually all cell types; they collectively make up over 30% of the genome and are individually 10 kilobases (kb) to 10 megabases (Mb) in size. LADs are gene poor (<2 genes/Mb, compared to ~8 genes/Mb on average in the human genome) and harbor features of heterochromatin such as di- and tri-methylated histone H3 lysine 9 (H3K9me2, H3K9me3). As a result, LADs constitute a repressive peripheral compartment of the nucleus. 

**Figure 1:**
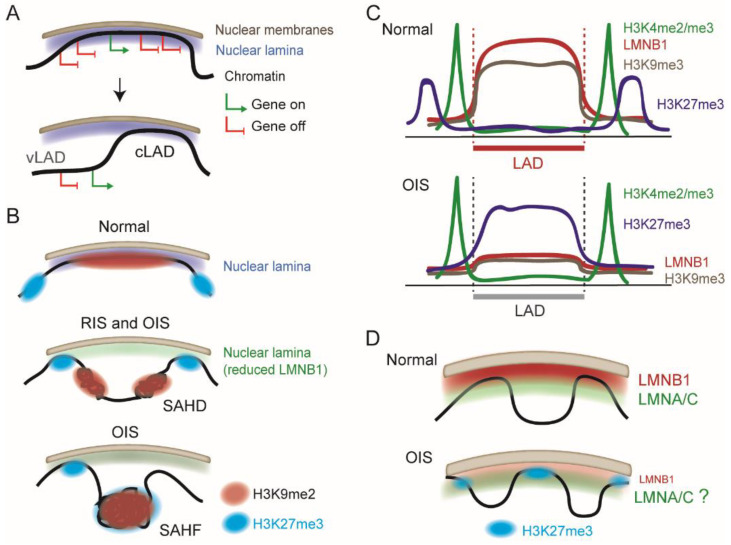
Lamina-associated domains (LADs) are dynamic features of peripheral chromatin architecture in senescence. (**A**) Chromatin association with the nuclear lamina via a LAD. LADs mostly harbor repressed genes, although occasional “escaper” regions contain expressed genes. While constitutive LADs (cLADs) are conserved during differentiation and between cell types, variable LADs (vLADs) are gained or lost; vLAD repositioning does not always concur with a change in gene expression. (**B**) Formation of SAHDs and SAHFs during replication-induced senescence (RIS) and oncogene-induced senescence (OIS). (**C**) Summary of aggregated profiles of LMNB1 and indicated histone modifications across LADs in normal cells and after OIS. (**D**) Speculative model of remodeling of nuclear lamina composition in OIS nuclei. Whereas loss of LMNB1 is documented, whether LMNA/C constitutes the main component of the lamina remains to be examined. OIS LADs are rich in H3K27me3 (see also panel B).

LADs are heterogeneous in their composition, dynamics, and transcriptional status (Figure 1A). Nevertheless, most LADs are well conserved across cell types and during differentiation [20,21,22,23]. Such constitutive LADs (cLADs) display the strongest LMNB1 enrichment, they are AT rich, enriched in long interspersed elements (LINEs), and strongly heterochromatic. Yet, cLADs may also harbor narrow euchromatic areas of lower LMNB1 contact frequency which are detectable by their enrichment in active histone modifications (such as H3K4me1, H3K4me3, and acetylated H3K27 [H3K27ac]) and expressed genes that escape the overall repressive context of the NL [24,25]. Moreover, during TGFβ-induced epithelial-to-mesenchymal transition (EMT), a fraction of LMNB1 has been shown to associate with euchromatic LADs containing active genes pertinent for EMT [26]. These are distinct from the euchromatic areas found in cLADs, but both could be of relevance in senescence and cancer genome architecture.

Variable (v)LADs arise during differentiation and therefore differ between cell types [19] (Figure 1A). vLADs emerge or disappear as entire domains or as edges of LADs that gain or lose contact with the NL [25], often but not always in line with the activation of cell type-specific genes within them [20,21,25,27,28]. Some vLADs are radially repositioned in the nucleus when losing NL association [29] but this is not always the case [27,30]. vLADs are smaller, more gene dense, and tend to be less heterochromatic than cLADs, with lower H3K9me2/me3 levels. Other subsets of LADs (often vLADs) harboring active histone modifications are bound by a nucleoplasmic pool of LMNA/C in the nuclear interior, where they may play a role in the regulation of chromatin organization, epigenetic marking of regulatory elements, and gene expression [31,32,33].

## 3. Chromatin Remodeling Elicited by Senescence

Senescence is the result of an irreversible cell proliferation arrest due to exhaustion of cellular replicative potential [2]. Replication-induced senescence (RIS) results from progressively shortening telomeres that are ultimately detected as irreparable DNA damage [3]. Senescence is also a response to various insults such as oxidative stress, DNA damage, chemotherapeutic agents, and stress signals elicited during development, wound healing, and regeneration. Oncogene activation also leads to a variation in the senescence process called oncogene-induced senescence (OIS) [2]. RIS and OIS lead to common characteristics including morphological changes, senescence-associated β-galactosidase accumulation, activation of cell cycle inhibition pathways via upregulation of P16*^INK4A^* and P21*^CIP1^*, reduced levels of LMNB1, and the senescence-associated secretory phenotype (SASP), a spectrum of damage-associated molecular patterns, bioactive lipids, and secreted pro-inflammatory molecules [34].

Senescent human fibroblasts exhibit broad chromatin re-organization manifested by alterations in 3D chromosomal contacts, DNA hypomethylation, distension and de-repression of centromeric repeats, and epigenetic reactivation of transposable elements that enhances chromatin accessibility [3,35,36]. RIS nuclei also frequently contain compacted chromosome arms, while both RIS and OIS nuclei display increased long-range chromosomal *cis*-interactions to form senescence-associated heterochromatin domains (SAHDs) rich in H3K9me3, LINEs, and LADs [37] (Figure 1B).

RIS and OIS, however, also differ in the chromatin architectural changes they elicit. A hallmark of OIS, which is rare in RIS, is the formation of senescence-associated heterochromatin foci (SAHFs), large masses of heterochromatin localized away from the NL [38] (Figure 1B). Microscopy and whole-genome chromosome conformation (Hi-C) data show that OIS nuclei display a much larger gain of chromosomal contacts than RIS nuclei, while losing intra-SAHD contacts and gaining long-range inter-SAHD interactions, forming the basis of SAHFs [37]. SAHFs display an atypical heterochromatin architecture, with H3K9me3 and heterochromatin protein 1 alpha HP1α/CBX5 enriched in the center and facultative heterochromatin marked by H3K27me3 localized in the outer layer [38,39,40] (Figure 1B). This geometry results from a spatial rearrangement of H3K9me3 and H3K27me3 domains rather than from epigenomic erasure and re-writing of these marks. The significance of SAHFs in the senescence phenotype in vivo remains unclear, however, as these structures have so far only been observed in cultured cells.

## 4. Repositioning of LADs during Oncogene-Induced Senescence 

The loss of LMNB1 characterizing senescence is probably at the origin of the formation of SAHFs away from the NL [41,42,43,44], effectively repositioning LADs. This LMNB1 loss is not uniform along the NL and preferentially occurs in regions richest in AT content and in H3K9me2/me3 [40,42,45,46]. OIS-induced repositioning of H3K9me2/me3 LADs is not accompanied by significant changes in the expression of protein-coding genes in these domains [40], consistent with the persistence of constitutive heterochromatin after the loss of NL interaction [45]. Of note, whether local areas of gene activity identified in cLADs in normal cycling or differentiated cells [24,25] retain their euchromatic and active states in an OIS context remains to be examined, but since they contain mostly cell type-specific genes [25], they may become repressed within the compact SAHFs, as part of the hijacking of cell fate induced by senescence.

The relationship between loss of NL interaction, SAHF formation, and gene expression changes in senescent cells is complex and may in part depend on the mode of senescence induction, early or late senescence stages, and the nature of genomic elements considered [47]. Repetitive DNA sequences can be transcriptionally deregulated in relation to their repositioning relative to the NL [47]. In OIS nuclei, telomeres preferentially relocalize to the NL [48] where, probably due to reduced lamin levels, they undergo structural rearrangement, loss of chromatin integrity, and dysfunction [49]. Supporting this view, loss of LMNA/C in mouse cells results in loss of telomere maintenance and reactivation of non-coding *Terra* transcription [50]. Downregulation of LMNB1 in OIS nuclei also leads to detachment of centromeric repeats from the NL, nucleoplasmic relocalization, satellite distension, and activation of these repeats, contributing to genomic instability [51]. These observations are in line with the increased expression level of not only L1 repeats [36,52], but also of a number of long terminal repeat (LTR) and DNA transposons, and several classical satellite and α-satellite repeats in late RIS cells [36,53,54]. Interestingly, SAHF formation may affect gene expression at more distal sites relative to SAHFs themselves. Indeed, protein-coding genes located 500–700 kb away from SAHFs have been found to be significantly downregulated [55]. In contrast, SAHF formation also brings together euchromatic protein-coding loci to enable their activation, including genes involved in cancer and inflammation responses (but not SASP genes) [37]. In fact, the large-scale genomic reorganization characterizing OIS also elicits expression of senescence-associated genes as a result of condensin-mediated transitions from repressive B compartments to active A compartments, and reinforcement of boundaries between these compartments [55]. 

As AT-rich domains lose LMNB1 interaction during senescence, smaller more GC-rich regions (so-called H-isochores) gain lamin interactions [45]. OIS-induced LADs are H3K27me3-rich [39,40], reflecting a reconfiguration of chromatin states in these domains (Figure 1B,C). In non-senescent cells, LADs are interspersed by H3K27me3 areas often found~50–200 kb away from LAD borders [15,56], or sometimes adjacent to the LAD [57]. Thus, H3K27me3-rich OIS-induced LADs may be positioned by an exchange of H3K9me2/me3 LADs with H3K27me3 domains in *cis*. One may however not formally exclude the possibility of a de novo H3K27 methylation of domains ending up as LADs in OIS nuclei.

The OIS-induced loss of LMNB1 at the NL expectedly leads to a shift in the ratio of LMNA/C to LMNB1 at the lamina, putatively favoring a proportion of LADs only bound to LMNA/C—a feature reported in hepatocarcinoma cells [30] and in normal human mesenchymal stem cells (our unpublished observations). Whereas LMNB1 preferentially interacts with H3K9me2/me3 domains, LMNA/C has been shown to be intertwined and interact with the polycomb repressor complex 2 containing the H3K27 methyltransferase EZH2 [58,59]. Consequently, a proportion of LMNA/C and H3K27me3 domains are in proximity. So, in line with the idea of an OIS-induced LAD shift, H3K27me3-rich LADs [39,40] could speculatively be predominantly bound to LMNA/C at the NL (Figure 1D). Exploring the fate of LMNA/C LADs during OIS-induced LAD repositioning may provide new mechanistic insights into these radial genome reconfigurations. This view is supported by the causal link between *LMNA* mutations and progeria, a premature aging syndrome characterized by a loss of heterochromatin at the NL [60,61].

GC content, along with replication timing, has been proposed to predict LAD rearrangement during OIS [37,45]; this is consistent with the higher GC content of H3K27me3 than H3K9me2/me3 domains [62], and with the higher gene density of OIS-induced LADs than bona fide cLADs [48]. Since genes within these H3K27me3-rich LADs overall retain their initial expression level [40], they are likely to be regulated in a lamin-independent manner, or alternatively, the NL of senescent cells loses its repressive influence through mechanisms that remain to be determined.

## 5. What Do We Know about LADs in Cancer?

**Figure 2:**
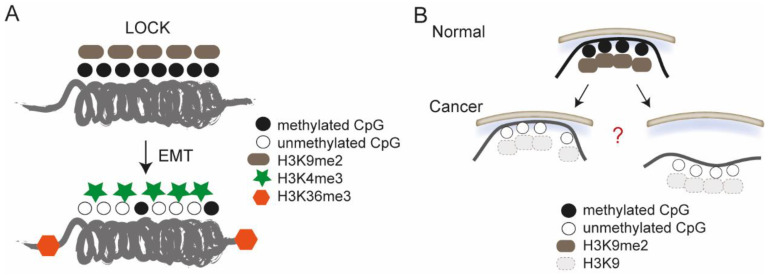
Epigenetic reprogramming of large organized chromatin K9 modifications (LOCKs)/LADs in cancer cells. (**A**) Epigenetic alterations targeted to LOCKs upon TGFβ-induced epithelial-to-mesenchymal transition (EMT). (**B**) LOCKs/LADs, naturally enriched in H3K9me2 and DNA methylated (top), lose their heterochromatic states in cancer (bottom). What is still unclear is whether cancer cell LADs are actually H3K9me2 poor and DNA hypomethylated, and whether LOCKs/LADs losing H3K9me2 detach from the nuclear lamina (bottom right) or are retained at the lamina as euchromatic LADs (bottom left).

Surprisingly little. Cancer cells are characterized by a reprogramming of their epigenome [63,64] and by spatial chromatin rearrangements exacerbated by variations in DNA content and deformations of the nuclear envelope [65]. These predict impairments in interactions of chromatin with the NL, but LADs have not systematically been examined in tumors. The role of LADs in development and differentiation has however raised the idea that they may also be involved in cancer initiation and progression. Domains the closest to LADs which have been characterized during TGFβ-induced EMT are so-called “large organized chromatin lysine modifications” (LOCKs). LOCKs have been initially identified in embryonic stem cells and in various cell types as domains rich in H3K9me2 and which strikingly overlap with LADs [66]. TGFβ-induced EMT elicits a reduction in H3K9me2 levels in these LOCKs and an increase in euchromatic histone modifications, including H3K4me3 in GC-rich areas along with H3K36me3 in regions flanking LOCKs [67] (Figure 2A). This reprogramming of LOCKs involves the H3K4/K9 demethylase LSD1 and occurs downstream of the remodeling of cell adhesion and cytoskeletal processes elicited by TGFβ [67].

A key epigenetic alteration in colorectal tumors is a partial loss of DNA CpG methylation in domains that strikingly coincide with LADs mapped in fibroblasts in a separate study [68] (Figure 2A) (anecdotally, this suggests that LADs from different cell types may be used as a proxy for LADs in tumor cells). In addition, metastatic foci in pancreatic ductal adenocarcinoma display broad reductions in H3K9me2 along with DNA hypomethylation in domains analogous to LOCKs [69], suggesting these effectively take place in LADs. What remains unknown is whether LOCKs/LADs losing H3K9me2 in cancer cells detach from the NL or are retained as LADs that have lost their heterochromatic state (Figure 2B).

LADs emerge as features of genome susceptibility to carcinogens. UV-induced DNA lesions in cultured cells prevail in LADs in fibroblasts and show enrichment at the nuclear periphery in 3D structural genome models [70]. This radial susceptibility mirrors the frequencies of (driver and/or passenger) mutations associated with melanoma, which in majority display the C > T conversion signature of UV-induced DNA lesions [71,72]. Thus, carcinogen susceptibility and mutation frequency go in pair with the association of the genome with the NL [70].

In addition to LADs, heterochromatin is tethered against nucleoli, as nucleolus-associated domains (NADs) [73]. NADs overlap with LADs, suggesting that the nucleolus and the NL are interchangeable scaffolds for heterochromatin. Increases in nucleoli size and number are hallmark features of many tumor types [74] and may enhance the nucleolar surface available for NADs, contributing to the remodeling of high-order chromatin architecture in tumor cells.

## 6. Re-KODing LOCKs in Tumor Cells?

Nuclear peripheral chromatin does not solely consist of LADs. Inter-LAD domains referred to as “H3K9me2-only domains” (KODs) have been shown in mouse embryonic stem cells to be enriched in cell type-specific enhancers [75]. KODs are also marked by H3K9me3 and are intriguingly also enriched in H3K27me3 (anecdotally questioning their denomination). Along with LOCKs/LADs, these domains could play a role in senescence or cancer chromatin architecture and gene regulation: loss of H3K9me2 in LOCKs [67,68,69] may also affect H3K9me2-only domains and favor transcriptional activity in these regions. Since KODs contain pluripotency genes [75], this would be compatible with the de-differentiation phenotype of tumor cells. Heterochromatin in these domains may also facilitate enhancer interactions with cognate genes in adjacent LADs, contributing to their upregulation, a feature in line with the euchromatinization of LADs/LOCKs in tumor cells. Alternatively, reconfiguration of peripheral chromatin in cancer cell nuclei may guide inter-LAD regulatory elements to tumor-specific LADs and conversely, may reposition normally in-LAD enhancers into inter-LADs.

## 7. Euchromatic LADs Associated with EMT

Whereas LADs associated with LMNB1 are typically heterochromatic in normal cells, interactions of LMNB1 with euchromatic LADs (eLADs) have been reported in TGFβ-induced EMT of normal mouse mammary epithelial cells [26]. Structurally, EMT is characterized by A/B compartment switches, with eLAD enriched in EMT A compartments. LMNB1 is enriched at the borders of topological domains showing increased border strength (i.e., enhanced stability), as well as at active genes relevant for EMT. Reducing LMNB1 levels alters EMT gene expression and phenotype, suggesting a role for LMNB1 in functionally stabilizing the genome during EMT in this model system [26]. It will be interesting to determine whether LMNB1 localization at active genes also occurs in human cellular models of EMT.

The results of Pascual-Reguant et al. are based on chromatin preparations enriched in euchromatic fragments, which favor enrichment of immunoprecipitated protein (including lamins) in these regions [31]. So, their findings do not exclude a co-existence of heterochromatin LMNB1 LADs, although EMT-associated A/B re-compartmentalization may reposition a fraction of these LADs into euchromatin. Bioinformatically, detection of lamin enrichment at genes vs. domains (LADs) is influenced by the domain or (narrow) peak calling algorithm used [76]. Applying both types of algorithms may unveil new patterns of lamin-genome interactions and provide additional insights into the regulation of transcriptional programs in normal, senescence, and cancer states.

## 8. Concluding Remarks

Observations highlighted in this review open the door to further investigations of LAD and inter-LAD peripheral chromatin dynamics, and of emerging roles of A- and B-type lamins in cancer initiation and progression [77,78,79,80,81]. Various tumors experience different stiffness constraints in vivo, which will expectedly modulate the ratio of A- to B-type lamins in the NL [82,83]. This is also probably exacerbated by the lower LMNB1 levels in tumors and may be affected by tumor type or stage, or tumor zone, between which lamin levels can vary [84,85]. A- and B-type lamins may also play distinct roles in cancer due to differences in their binding partners [16], the distinct polymers they form and their distinct localizations in the NL [86], the LADs they associate with [30], and lastly, the LMNA/C-specific nucleoplasmic soluble pool [31,87]. Altered LMNA/C levels in cancer may differentially affect these sub-nuclear pools and confer phenotypes not mediated by changes in levels of B-type lamins in the NL. A role for LMNA/C in cancer is receiving increasing attention. Evidence implicates LMNA/C in preserving nuclear integrity in ovarian epithelial cancer subtypes [88] and non-small-cell lung carcinoma cells [79]. Accordingly, lower LMNA/C levels in invasive breast cancer cells confer increased nuclear deformations enabling enhanced cell invasiveness [89]. Recent implications of LMNA/C in the p53/p16*^INK4A^* senescence pathway [80] suggest an interplay between chromatin and signaling functions modulated by A-type lamins which will be worth investigating more closely.

It will be informative to characterize LAD dynamics and composition in models of cancer progression or in cancer cells that resist chemotherapeutic agents, as well as the impact of senescence-inducing cancer therapies on LADs. A challenge in investigating lamin–genome interactions in cancer, however, is the expected large variability between tumor type, stage, site, and composition, and between cells within a specific tumor zone. Genomic rearrangements may further enhance LAD variability, which may be greater within a tumor than between different normal cell types. Tumors also harbor several cell types that unless discriminated will contaminate (epi)genomic analyses of the actual cancer cells. Interpretable models of cancer progression of known genome content will be beneficial to mechanically investigate the dynamics of chromatin rearrangements at the nuclear periphery.

Future investigations combining structural and fluorescence imaging [86,90], with rapidly evolving genomics approaches pertaining to single-cell cancer genomics [91], will increase our understanding of how the nuclear envelope and chromatin architecture contribute to regulating cell fate in health and disease. Deep learning methods aimed to recognize nuclear morphologies will in addition enhance cancer diagnosis accuracy [92]. Many genetic, epigenomic, and structural factors influence genome reorganization in cancer cells. Being able to distinguish the contribution of LADs to cancer cell phenotypes amidst these factors remains a major challenge.
